# Economic barriers and gaps to reach the desirable consumption of salt, sugar, and fat in Iran: a qualitative study

**DOI:** 10.1186/s41043-023-00348-8

**Published:** 2023-01-30

**Authors:** Mohammad Amerzadeh, Amirhossein Takian, Hamed Pouraram, Ali Akbari Sari, Afshin Ostovar

**Affiliations:** 1 Social Determinants of Health Research Center, Research Institute for Prevention of Non-Communicable Diseases, Qazvin University of Medical Sciences, Qazvin, Iran; 2 Department of Health Management, Policy and Economics, School of Public Health, Tehran University of Medical Sciences (TUMS), Poursina Ave, Tehran, Iran; 3 Department of Global Health and Public Policy, School of Public Health, Tehran University of Medical Sciences (TUMS), Tehran, Iran; 4 Heath Equity Research Center (HERC) – TUMS, Tehran, Iran; 5 National Center for Health Insurance Research, The Iranian Health Insurance Organization, Tehran, Iran; 6 Ommunity Nutrition Department, School of Nutritional Sciences and Dietetics, Tehran University of Medical Sciences (TUMS), Tehran, Iran; 7 Osteoporosis Research Center, Endocrinology and Metabolism Clinical Sciences Institute, Endocrinology and Metabolism Research Institute, Tehran University of Medical Sciences (TUMS), Tehran, Iran

**Keywords:** Economic barriers, Non-communicable diseases (NCDs), Fat, Sugar, Salt, Iran

## Abstract

**Background:**

Non-communicable diseases (NCDs), also known as chronic diseases, specifically cardiovascular diseases (CVD), cancers, respiratory diseases, and diabetes are the main reason for more than two-thirds of global deaths, in which the unhealthy diet is one of the primary risk factors. The golden solution to reducing obesity and CVD linked to an unhealthy diet is to reduce calories, salt, sugar, and fat intake. Besides, activities highlighting lifestyles that contain healthy diets usually focus on reducing salt, sugar, and saturated fat consumption. As a result, the researchers aimed to study the gaps and economic barriers to recommended consumption of salt, sugar, and fat in Iran, based on WHO recommendations.

**Methods:**

This is a qualitative study. We conducted semi-structured and in-depth interviews with 30 stakeholders, including academics, experts, and key informants in different sectors from December 2018 until August 2019 in Tehran, Iran. We used a purposeful and snowball sampling method to select participants. All interviews were transcribed verbatim and thematically analyzed using MAXQDA 11.

**Results:**

Economic problems and inflation in Iran caused people to eat more unhealthy foods, while a healthy diet consumption was reduced due to higher prices. Unfair political sanctions imposed on the country caused economic pressure and adversely affected family nutrition. Worse still, despite legal bans, advertising unhealthy foods via media, mainly to generate revenue, encouraged more consumption of unhealthy food. The lack of targeted subsidies and failure in tax legislation and implementation related to the unhealthy products deteriorated the conditions.

**Conclusion:**

Some economic barriers have hampered plans to reduce salt, fat, and sugar consumption in Iran. Fundamental reforms in the tax and subsidy system are required to improve people’s eating habits. In particular, citizens’ income that has been continuously shrinking due to economic conditions, imposed sanctions, and the inevitably high inflation needs to be addressed urgently. Unless the government of Iran deals with the economic barriers to healthy nutrition, the pathway for implementing the national action plan for prevention and control of NCDs toward a 30% mortality reduction due to NCDs by 2030 looks unlikely to reach.

## Background

Non-communicable diseases (NCDs), also known as chronic diseases, specifically cardiovascular diseases (CVD), cancers, respiratory diseases, and diabetes are the main reason for more than two-thirds of global deaths (38 million deaths out of 56 million annual deaths). Four-fifths of NCDs occur in low- and middle-income countries (LMICs), and a third of deaths from NCDs are people under 60 years [1–3]. By 2030, the annual NCDs mortality is predicted to increase to 52 million [4]. Let alone, the COVID-19 pandemic is likely to dramatically increase the burden of NCDs [5]. The golden solution to reducing obesity and CVDs linked to an unhealthy diet is to reduce calories, salt, sugar, and fat intake [6]. The lack of healthy foods such as fruit, vegetables, whole grains, seafood, nuts, and pulses, combined with the high consumption of processed food with high sugar, salt, and trans fat, contribute to this health problem across the world [7].

NCDs can lead to catastrophic, long-term economic consequences for people and their families, in particular, in resource-poor conditions. Many NCD patients are faced with either treatment, thereby making their families poor or not receiving treatment, which has adverse health and economic conditions for the individual and the household [8]. People with low economic conditions in high-income countries have more possibility to have a higher NCDs disease burden than people with high economic conditions [1, 9]. Insurance coverage, health-service support, prevention programs availability and efficiency, level of disability, and family and community networks are some determinants that can lead to different NCDs’ consequences [8, 10].

NCDs are also linked with poverty and might cause inequality within and across countries. Catastrophe expenditures from NCD-related medical care are very likely even among people with insurance coverage. Ample evidence demonstrates high health dividends from investments in the prevention and control of NCDs, in high-, middle-, and low-income countries alike, which can also assist economic growth [11].

Risk factors of NCDs are classified into modifiable (behavioral), metabolic, and physiological risk factors. Modifiable risk factors include tobacco use, physical inactivity, unhealthy diet, and harmful alcohol use, among which poor diet is the biggest contributor to NCD’s burden [12]. An efficient strategy to reduce NCDs is focusing on reducing these risk factors, early detection, and appropriate treatment of NCDs [13]. Globally, high consumption of processed food with extra amounts of sugar, salt, saturated and trans fats are combined by low consumption of healthy foods like fruit and vegetables, whole grains, nuts, pulses, and seafood [14]. The loss due to NCDs in economic production is predicted to be $47 trillion worldwide [15]. The economic expenditure of unhealthy diets and inadequate physical activity in the European Union (EU) is €1.3 billion annually [16]. The benefits of a 1 g of salt intake daily reduction per person lead to the prevention of 4,147 annual premature deaths in the UK and savings of £ 288 million [17].

Policymakers mainly target obesity and cardiovascular diseases by decreasing the extra intake of calories, sugar, salt, and saturated fats [18]. Activities highlighting lifestyles that contain healthy diets usually focus on reducing the consumption of salt, sugar, and saturated fat. Policymakers mainly have targeted obesity and cardiovascular diseases by decreasing the extra intake of calories, sugar, salt, and saturated fats [18]. Iran is in a transition era due to population aging, changes in disease risk factors, and the replacement of communicable diseases by NCDs [19]. NCDs are the leading cause of death in Iran, causing 83% of total death in 2019, with an unhealthy diet as the leading risk factor [20]. In the top ten leading causes of death in Iran, eight are categorized as NCDs [21]. Several risk factors are noticeable in Iran due to unhealthy dietary habits, i.e., high salt consumption and saturated oil, low physical activity, and high tobacco use [22]. Diverse interventions are among the primary health sector interventions in Iran to reduce the incidence and mortality of NCDs, namely improving unhealthy diet and reducing salt, sugar, and fat consumption at both industry and population levels [23]. Studies on the relationship between economic status and NCDs in LMICs are scarce. This study aims to identify the gaps and economic barriers to the WHO recommendations for the consumption of salt, sugar, and fat in Iran to help the policy makers and healthcare managers control NCDs better and reach the target in the long run.

## Methods

### Study design and data collection

This is a qualitative study that used semi-structured, in-depth, and face-to-face interviews with relevant key informants from December 2018 until August 2019 in Tehran to explore the economic barriers to reducing salt, sugar, and fat consumption in Iran. The researchers used this method since open-ended questions invite participants to describe the goals, process, consequences, and challenges.

### Setting and sampling

The researchers selected 30 participants through purposive snowball sampling of stakeholders, i.e., academics, experts, and key informants with high experience and knowledge in policymaking, health management, health promotion, and planning. Researchers chose interviewees who could provide valuable information, were available and had relevant information. A few interviewees were not keen to take part in the research and they were replaced by other participants from the relevant sector. They provided the interviewees with an information sheet that described the study's aims, the interviewees’ roles, the strategies for data security, anonymity and confidentiality, and the voluntary nature of participation. Participants ranged from 35 to 78 years old, including nine females and 21 males. They were from various sectors, including MoHME, Ministry of Agriculture, Ministry of Industry, Mine and Trade, Ministry of Education, Municipalities, National Standard Organization, Ministry of Economic Affairs and Finance, Islamic Republic of Iran Broadcasting (IRIB), Planning and Budget Organization and Iranian Academy of Medical Sciences (AMS) (Table 1).

**Table 1:** The characteristics of interviewees

Age	Ranged from 35 to 78 years old
Gender	9 females and 21 males
Location	Ministry of Health and Medical Education (MoHME)
	Ministry of Agriculture
	Ministry of Industry, Mine and Trade
	Ministry of Education
	Municipalities
	Standard Organization
	Ministry of Economic Affairs and Finance
	Islamic Republic of Iran Broadcasting
	Planning and Budget Organization
	Iranian Academy of Medical Sciences
Participants’ background	A mixture of policymakers, health system managers, university professors, and specialists

The research team developed a generic interview guide comprising 15 main questions based on the literature review and the study objectives. The researchers earlier published the interview guide elsewhere [24]. All interviews were conducted in the participants’ work place. Interviews lasted 30–90 min and were digitally recorded and transcribed verbatim. The corresponding author (AT) conducted the first interview in the presence of the first author (MA) and subsequently, the first author (MA) conducted other interviews, approved and secondary analyzed by AT. Notes were made during the interviews, and the place, date, time, and other significant issues were all recorded. The researchers conducted interviews until data saturation, so no new themes were identified. Characteristics of interviewees are described in Table 1:

### Data analysis

The first author (MA) began a preliminary analysis of each interview immediately after the end of each interview and adopted the inductive–deductive approach for data analysis [25, 26]. First, each interview was thematically analyzed and open codes emerged through an inductive approach, which was then grouped into clusters. The team produced broader categories and subcategories from these concepts via constant comparison or verification. The outcome was a matrix of categories and subcategories used for developing theoretical or conceptual frameworks. First and corresponding authors conducted the categorization, with all authors revising and approving the entire process. Further, the research team asked other researchers familiar with the analysis of qualitative research to check some of the interviews, codes, and themes to revise their validity. To increase credibility, researchers sent some transcripts, categories, subcategories and codes to selected interviewees to obtain their approval about the accuracy of the interpretations [27, 28]. The researchers carried out the qualitative content analysis using deductive and inductive approaches for data analysis, facilitated by MAXQDA 11 software into MAXQDA 11 (VERBI software, Germany), for consistent data analysis, processing, management, and comparison of the results at various levels.

## Results

The researchers emerged five categories in our analysis. Table 2 summarizes the main categories of our findings:

**Table 2:** Economical barriers to standard consumption of salt, sugar, and fat in Iran

Theme	Category	Examples of economic barriers
Economical factors	The adverse impact of sanctions on people’s choice in diet	Challenging the government to implement some policies For households to provide the family’s food basket
	Inadequate income	Insufficient revenues lead to purchasing unhealthy and cheap food
	Inappropriate media advertisement	Islamic Republic of Iran Broadcasting (IRIB) and municipalities’ significant portion of revenue through advertising harmful products
	Undesirable subsidy system	The subsidy system deviated from its initial purposes and could not reach to aims
	Failure in implementing tax policies	Lack of a system that imposes a tax on unhealthy products to increase the price

### The adverse impact of sanctions on people’s choice in diet

Economic shortcomings were identified as one of the primary causes of the increasing unhealthy diet in Iranian society. Healthy foods are more expensive; hence, as the economy deteriorates, people tend to eat products with higher fat, sugar, and salt. Some interviewees pointed out to unilaterally imposed sanctions due to the US withdrawal from the Joint Comprehensive Plan of Action (JCPOA) in 2018, which resulted in economic deterioration and households’ difficulties to provide the family’s healthy food baskets:

Nutrition highly depends on the economy. Implementation of related policies requires economic stability. The US withdrawal from JCPOA and imposing sanctions has made implementing some nutrition policies a little difficult. (Policy maker 2)

Therefore, sanctions are considered the first economic barrier to a healthy diet.

### Inadequate income

Enough consumption of healthy food such as fruits and vegetables (five servings per day) can reduce the intake of salt, sugar, and fat. Nonetheless, most people do not follow these recommendations. Therefore, the unhealthy diet has been rising for various reasons, including inadequate income, particularly among citizens in low-paid jobs:

When our government does not give salaries based on the minimum necessities, it causes many problems. For example, if a worker with a family of four wants to eat healthy foods, he has to use up half of his revenue, whereas diet is 25% of the basket of goods. Housing is crucial, so the family has to spend less on food. The government must modify the salary system so that people can afford to buy a healthy diet. (Faculty member 4)

Households deteriorating economic status has shifted the priority of many families to only provide essential energy, while food quality gets the second priority:

The most critical challenge is the price. When people's economic situation deteriorates, the first things they eliminate are milk, fruit, and vegetables. (Policy maker 2)

Some experts predicted that the increasing trend of unhealthy diet and its consequences would rise in the future:

Nowadays, the status of the country is specific. We are under heavy sanctions by the U.S. and in the economic war. The troubles like obesity will rise in the next generation. (Policy maker 9)

As a result, sufficient income can resolve many problems related to the unhealthy diet.

### Inappropriate media advertisement

One problem associated with sugar, salt, and fat consumption is the high consumption of harmful products in which economic factor plays an important role; the IRIB and municipalities derive a significant portion of their revenue through advertising the harmful products. One expert from the municipality explained that they advertise these products, although it is prohibited:

Its instruction is not enforced for several reasons. One reason is that some companies rent the billboards and they need to compensate for their costs and unhealthy products payment is tempting. (Municipality expert 20)

Besides, there are enough laws for media management. Nevertheless, many of them are not implemented efficiently:

There is a law for all advertisements, but it is not enforced. The IRIB expressed that harmful products pay very well, and we need to compensate for our expenditures. If the MoHME pays more money, we will advertise healthy products. (Faculty member 4)

Many experts expressed that the main cause of these problems is economic difficulties which resulted in insufficient budget or revenue for these organizations:

The economy is sick, and the media is working for survival. As a result, the University Entrance Exam and harmful products take priority because they pay more for advertising. (Health expert 6)

Therefore, solving the revenue of media can be another facilitator.

### The undesirable subsidy system

Another problem is inappropriate implementation of the targeted subsidies law, designed to improve jobs creation and help industries promote public health and produce healthy products. Implementation of this law was launched in 2010 to replace subsidies on food and energy with targeted social assistance, in line with a Five Year Economic Development Plan. Its fundamental goal was to rejuvenate the economy and increase productivity:

Another problem is the wrong subsidy system. It seems that subsidies are applied to meet government goals and generate revenue. (Health expert 15)

Besides, the interviewees accused the government of not utilizing the targeted subsidies to enhance access to healthy foods. The strategies for allocation of targeted subsidies toward healthy products were branded and not feasible to be executed:

Considering policies to increase access (both physical and economic access) to vegetables and fruit, beans, bread, and whole grains are also important. However, you cannot see them in many policies like the National Action Plan for the Prevention and Control of NCDs. (Policy maker 1)

Some interviewees also accused the Targeted Subsidies Law of 2010 deviated from its initial purposes, which could not reach its aims related to the health system:

In the Targeted Subsidies Law, one goal is to improve the nutritional pattern and access to a healthy diet. But the targets of this plan were deflected and the consumption pattern did not change. (Health expert 13)

An inefficient subsidy system is the fourth barrier to promoting a healthy diet.

### Failure in implementing tax policies

Many countries apply the tax system to promote healthy products, e.g., foods. Imposing a sin tax on unhealthy products will increase their price and reduce demand. Despite the successful experience of some countries in implementing such policies for products with a high level of salt, sugar, and fat, some participants complained about insufficient implementation of these policies in Iran:

We failed in taxation because it requires the law and the cooperation of other organizations. Unfortunately, we couldn’t do anything positive, even taxing cigarettes, which is a harmful product. (Policy maker 26)

Therefore, implementing the right tax policies can solve many financial problems of an unhealthy diet.

The economic expenditure on unhealthy diets can lead to an increased burden of diseases and the consequent cost for families and the health systems. Paying particular attention to people’s financial status and designing specific innervations to promote a healthy diet can lead to healthier communities.

## Discussion

This study aimed to identify the gaps and economic barriers to the desired consumption of salt, sugar, and fat in Iran, based on WHO recommendations to provide evidence toward improving the related policies. Our findings can help the government and various stakeholders, we envisage, to initiate tailored interventions to improve economic conditions that might facilitate selection of a healthier and reduce the consumption of foods with excessive salt, fat, and sugar.

Our findings revealed that because of economic problems and high inflation in Iran, people’s tendency has increased to eat more unhealthy foods with higher levels of fat, sugar, and salt. At the same time, consumption of healthy diet has reduced, mainly due to their higher prices, particularly among the poor and vulnerable households. Currently, the priority of many families is only energy supply. Low-income families spend average 30% of their income on housing costs. Roughly 30% of income is essential to eat healthily, so families must spend almost 60% of their income on food and accommodation. Therefore, little room remains to afford other necessary expenses, i.e., bills, transportation, education, and medical expenses [29]. Another study also found that the withdrawal of the US Government from the JCPOA and consequent sanctions against Iran in 2018 led to unusual inflation in the food market. The prices of many food items rose over 50% the year after the sanctions. The highest inflation was in fruits, vegetables, and meat products, and the least was in white bread and oils [30]. As a result, many families were left with no choice than reducing their spending on healthy food and allocating it to other costs. A UK study to investigate eating habits and health conditions among people with lower income revealed less consumption of quality diet and more use of energy-dense products like processed meat, full-fat milk, sugar, and soft drinks compared to those with higher incomes [31]. Therefore, the government needs to formulate policies to control inflation, aiming to help the vulnerable population and promote a healthy diet in the country.

The ongoing unilateral sanction imposed on Iran and its economic consequences have also jeopardized family nutrition among many households in Iran. One complex dimension of sanctions is the relationship between the economic conditions and health consequences in the country [32]. Our study backs the claims that economic sanctions can result in inflation, reduced household spending, and problems in the health insurance system function [33]. Other studies also highlight that sanctions can reduce people’s access to necessities like nutritious food and medical care via deteriorating economic situations [30, 34]. Studies revealed that sanctions end up in poor diet, increased infant mortality, and decreased access to food [35]. Many studies have reported the impact of sanctions on health indicators, including mortality indicators, life expectancy at birth, healthy diet, and drinking water quality [36, 37]. Studies also show that imposing sanctions led to sudden declines in Iran's currency value, which in turn resulted in a sharp increase in the price of food, and a severe rise in unemployment, reducing household expenditures by using less quality and quantity of food. [38, 39]. The situation even worsened during the COVID-19 pandemic [40]. One indirect effect of sanctions was growing global concern about HIV/AIDS among vulnerable populations, e.g., women and children [41]. We advocate government take any necessary step to support people from the adverse effects of sanctions, ease the consequence, adopt the right economic policies, and reform salary payments.

Further, advertising unhealthy foods via media causes more consumption of them. A systematic review found that food advertisements directly affected kids’ knowledge of diet, selections, shopping behaviors, consumption patterns, and nutritional health [42]. One reason is the inappropriate system of generating revenue in Iran's media; harmful advertising products generate high revenue for the IRIB and the municipalities. In the USA, the annual expenditures of the food industry on advertising and promotions were over $30 billion in 2004, higher than any other industry [43]. In Iran, the MoHME has inadequate power and authority to deal with these industries, nor can it afford healthy advertising. We advocate the meaningful enforcement of the parliament law to allocate 1% of various public entities’ income toward training programs in the IRIB.

The targeted subsidies law has been implemented since 2010 in Iran, aiming to improve equity across Iranian society. The reform was expected to allocate the subsidies released from food and energy toward creating more jobs and helping industries promote public health and produce healthy products. Nevertheless, it faced many obstacles due to its flawed formulation, inappropriate implementation, and unregulated evaluation [44]. In many countries, governments impose a tax on products that negatively impact health while subsidizing healthy goods, so people in lower socioeconomic groups can afford to buy them. For example, cigarettes, soft drinks, and alcohol are highly taxed, and the generated revenue is spent on consuming vegetables, fruit, dairy, and fish. Mainly due to inadequate multi-sectoral cooperation, imposing a tax on unhealthy products has faced challenges during legislation and implementation. The gap has fueled some opposition from the industries to resist the implementation of tax law. A study found that an excise tax of around 13% on sweetened beverages in the Philippines may enhance population health. It revealed that the tax will most affect the richest quintiles [45]. In Iran, the government subsidizes sugar and oil, which can exacerbate the burden of NCDs and obesity. Another study in the UK also suggests that salt is cheap, and a 40% tax may lead to at least a 6% reduction in consumption [46]. We strongly advocate the government's work on taxing food products with a high level of sugar, salt, and fat and subsidizing healthy goods to adjust the supply and demand.

### Rigor of study

To the best of our knowledge, this study is one of the first to identify the gaps and economic barriers to recommended consumption of salt, sugar, and fat in Iran. Despite our efforts, a few interviewees were not keen to participate in our research. Nevertheless, the in-depth interviews with different stakeholders allowed us to collect good data sources. We conducted this study before the COVID-19 pandemic. Therefore, peoples’ diet might have changed due to economic conditions and food accessibility in particular in vulnerable citizens.

## Conclusions

An unhealthy diet is the leading cause of NCDs in Iran, addressing which is essential to reach the goals of the national action plan for the prevention and control of NCDs and reducing deaths attributed to NCDs. The deteriorating economic situation has led Iran to face obstacles in reducing salt, fat, and sugar consumption. Difficult decisions to reform the tax and subsidy system are pivotal to addressing the increasing intake of unhealthy foods in Iran. In particular, citizens’ income that has been continuously shrinking due to economic conditions, imposed sanctions and the inevitable high inflation needs to be addressed urgently. Implementing tailored policies to improve people's economic status, including increasing salaries and purchasing power, reforms in the country's tax and subsidies system, and better media management are essential to address this challenge. Unless the government of Iran deals with the economic barriers to healthy nutrition, the pathway for implementing the national action plan for prevention and control of NCDs toward a 30% mortality reduction due to NCDs by 2030 looks unlikely to reach.

## Data Availability

The datasets used and/or analyzed during the current study are available from the corresponding author upon reasonable request. The entire dataset is in the Farsi language.

## Abbreviations

NCDs: Noncommunicable diseases
JCPOA: Joint Comprehensive Plan of Action
LMICs: Low- and middle-income countries
MoHME: Ministry of Health and Medical Education
TUMS: Tehran University of Medical Sciences
MA: Mohammad Amerzadeh
AT: Amirhossein Takian
MOHME: Ministry of Health and Medical Education
IRIB: Islamic Republic of Iran Broadcasting
HP: Hamed Pouraram
AKS: Ali Akbari Sari
AO: Afshin Ostovar
Ph.D.: Philosophy of Doctrine
